# Is mild dehydration a risk for progression of childhood chronic kidney disease?

**DOI:** 10.1007/s00467-024-06332-6

**Published:** 2024-04-18

**Authors:** Amelia K. Le Page, Evan C. Johnson, Jason H. Greenberg

**Affiliations:** 1 Department of Nephrology, Monash Children’s Hospital, Clayton, VIC Australia; 2 Department of Pediatrics, School of Clinical Sciences, Faculty of Medicine, Nursing and Health Sciences, Monash University, Melbourne, VIC Australia; 3 Division of Kinesiology & Health, College of Health Sciences, University of Wyoming, Laramie, WY USA; 4 Section of Nephrology, Department of Pediatrics, Yale University School of Medicine, New Haven, CT USA; 5 Department of Internal Medicine, Clinical and Translational Research Accelerator, Yale University, New Haven, CT USA

**Keywords:** Chronic kidney disease (CKD), Children, Dehydration, Underhydration, Hypohydration

## Abstract

Children with chronic kidney disease (CKD) can have an inherent vulnerability to dehydration. Younger children are unable to freely access water, and CKD aetiology and stage can associate with reduced kidney concentrating capacity, which can also impact risk. This article aims to review the risk factors and consequences of mild dehydration and underhydration in CKD, with a particular focus on evidence for risk of CKD progression. We discuss that assessment of dehydration in the CKD population is more challenging than in the healthy population, thus complicating the definition of adequate hydration and clinical research in this field. We review pathophysiologic studies that suggest mild dehydration and underhydration may cause hyperfiltration injury and impact renal function, with arginine vasopressin as a key mediator. Randomised controlled trials in adults have not shown an impact of improved hydration in CKD outcomes, but more vulnerable populations with baseline low fluid intake or poor kidney concentrating capacity need to be studied. There is little published data on the frequency of dehydration, and risk of complications, acute or chronic, in children with CKD. Despite conflicting evidence and the need for more research, we propose that paediatric CKD management should routinely include an assessment of individual dehydration risk along with a treatment plan, and we provide a framework that could be used in outpatient settings.

**Graphical Abstract:**

**Figure:**
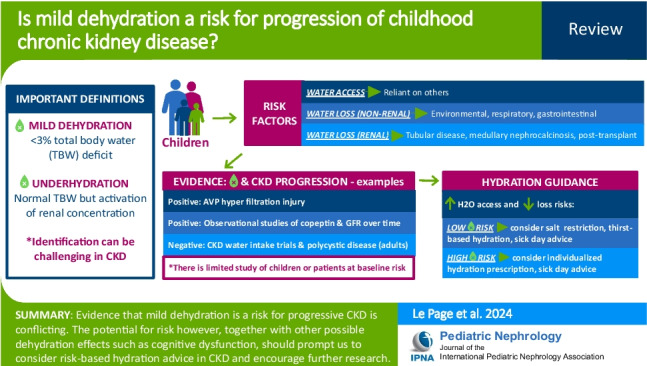
A higher resolution version of the Graphical abstract is available as Supplementary information

**Supplementary Information:**

The online version contains supplementary material available at 10.1007/s00467-024-06332-6.

## Introduction

Water intake has been encouraged in various kidney diseases, and in 2015 hydration was a focus of World Kidney Day. At the time, however, research had not clearly characterised the relationship between hydration and kidney health. Additional research has since been undertaken aiming to clarify this relationship, and whether an optimal hydration state can be defined.

In paediatric kidney disease, adequacy of hydration may be especially relevant. In some scenarios this population may be at higher risk of dehydration, for example where thirst response or access to water is restricted in infants, or in children with an oral aversion. Paediatric CKD patients often include those with impaired urine concentrating capacity which can lead to polyuria. This may increase risk of dehydration especially in the setting of decreased oral intake, additional fluid losses such as with concurrent gastroenteritis, or in hot weather. These issues may become exacerbated in the era of climate change.

The primary objective of this review is to summarise the literature on dehydration risk factors and consequences for children with CKD, with a particular focus on the effects of chronic mild dehydration or underhydration, and whether this may contribute to CKD progression. To inform this review we first provide readers with a relevant update on the physiology of water homeostasis and clinical assessment of hydration state. Our final objective is to provide a framework for assessment of dehydration risk and optimisation of hydration state.

## Physiology of water homeostasis

Homeostatic mechanisms are constantly working to balance water intake and losses and to maintain body compartment water distribution. To achieve this, plasma osmolality (the number of dissolved particles per kg of water) is finely balanced in the range of 285–295 mOsm/kg. Dehydration-associated hyperosmolality is defended by thirst-triggered water intake and arginine vasopressin (AVP)-mediated water resorption from the urinary space. Plasma osmolality increases of as little as 1% stimulate synthesis and secretion of AVP from the pituitary gland [1]. Thirst responses are classically perceived at a slightly higher osmolality threshold [2]. Hypovolaemia, regardless of plasma osmolality, independently stimulates both AVP release and thirst [2, 3].

**Fig. 1:**
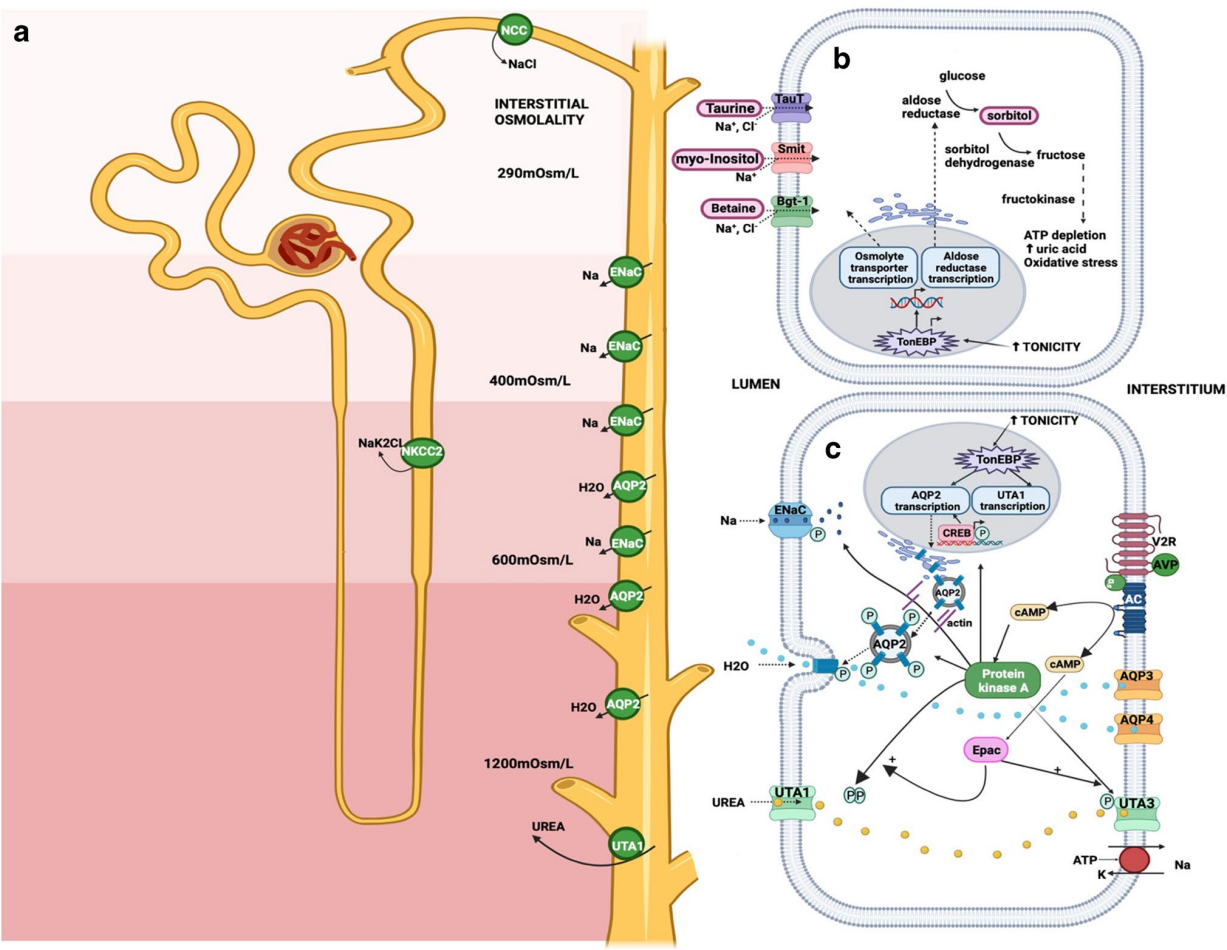
**A** Juxtamedullary nephron — key sites of V2R-mediated regulation urine concentration. **B** Schematic of tubular cell osmolyte effects relevant to urine concentration and CKD. **C** Schematic of collecting duct tubular cell - effects relevant to urine concentration. **1A**. AVP activation of V2 receptors leads to channel activity that contributes to medullary hypertonicity and water reabsorption [4–6]. The receptors indicated here all give rise to substantial urinary water loss and dehydration in rodent knock-out. Green circles represent a channel with movement of water or electrolytes from the tubule lumen into the interstitium via the cell. **1B**. Tubular cell osmolyte regulation. Synthesis and reabsorption of osmolytes is altered in response to changes in osmolality [7]. Increased intracellular osmolality increases activity of the nuclear factor TonEBP [8]. TonEBP increases transcription of osmolyte transporters leading to reabsorption of these compounds from the tubular lumen. TonEBP also increases transcription of aldose reductase that converts intracellular glucose to sorbitol which also acts as an osmolyte [9]. In the proximal tubule the enzyme fructokinase acting on sorbitol may contribute to damaging intracellular changes [10]. **1C**. Processes contributing to water reabsorption within tubular cells of the inner medullary collecting duct. As reviewed in [1], AVP binds to the V2R receptor and stimulates adenylate cyclase with production of cAMP. Downstream effects include stimulation of aquaporin 2 transcription, and AQP2 phosphorylation. Aquaporin 2 vesicles are steered to the luminal membrane by changes to the actin cytoskeleton, with channels endocytosed into the membrane, allowing water transport. Water traverses the cell and exits the basal membrane into the intersitium via constitutively expressed AQP3 and AQP4 channels. AVP via protein kinase A phosphorylates and activates the Urea channels UTA1 and UTA3 that also allow urea transport through the cell and into the interstitium, to set up the medullary concentrating gradient that promotes water reabsorption [4]. Epac is a protein kinase A-independent promotor of urea channel ac va on [11]. AVP also promotes EnaC activation and sodium reabsorption [6]. TonEBP is a non-AVP dependent stimulator of urine concentration via AQP2 and UTA1 channel transcription [8, 12]. *Created with BioRender.com*. *Na*, sodium; *Cl*, chloride; *K*, potassium; *H2O*, water; *mOsm/L*, milliosmoles per litre; *NKCC2*, sodium potassium chloride cotransporter; *NCC*, sodium chloride symporter or thiazide sensitive NaCl cotransporter; *ENaC*, epithelial sodium channel; *AQP2,3,4*, aquaporin channels; *UTA1*, *UTA3*, urea transporters; *ATP*, adenosine triphosphate; *Epac*, exchange proteins directly ac vated by cAMP; *cAMP*, cyclic adenosine 3,5 monophosphate; *AC*, adenylate cyclase; *P*, phosphate; *AVP*, arginine vasopressin; *V2R*, vasopressin type 2 receptor; *CREB*, cAMP-response element binding protein; *TonEBP*, tonicity-responsive enhancer binding protein; *TauT*, taurine transporter; *Smit*, sodium myo-inositol transporter; *Bgt-1*, betaine GABA transporter

With a normal glomerular filtration rate (GFR), our kidneys have evolved to conserve as much as 99% of filtered water. Most water is reabsorbed in the proximal tubule by osmotic drag linked to sodium transport. The key site of regulation of water reabsorption is more distal, at the collecting duct triggered by AVP action at basolateral membrane vasopressin 2 receptor (V2R) (Fig. 1A,C). V2R activation promotes aquaporin (AQP) 2 channel translocation to the luminal membrane, allowing water movement through the cell, which then exits into the interstitium via constitutively expressed basolateral AQP3 and AQP4 channels. Ultimately, when the osmotic gradient returns reabsorbed water to the capillaries, plasma osmolality is reduced, and homeostasis restored [1]. An important requirement for this process is the osmotic pressure set up by a highly concentrated medullary interstitium secondary to V2R-stimulated sodium and urea reabsorption (Fig. 1A) [4]. Through these mechanisms, the urine concentration can reach a maximum of 1200 mOsm/kg [1].

Hypovolaemia associated with dehydration also leads to many other linked homeostatic responses including activation of the renin–angiotensin–axis and sympathetic nervous system. These responses have been well defined, especially when associated with acute isotonic hypovolaemia, and are triggered by intravascular baroreceptors [2, 13]. In the case of hypertonic hypovolaemia (i.e. with water loss), there are also osmolality triggers of these responses. For renin, the osmolality trigger is indirect via AVP at the juxta-glomerular apparatus [14], and for sympathetic responses it is direct via brain osmoreceptors [15]. These pathways lead to vasoconstriction and sodium reabsorption, and they also contribute to thirst responses, thus all helping to defend the extracellular volume.

Amongst the countless additional processes that help maintain osmotic homeostasis are the mechanisms that ensure cells maintain their function in a state of dehydration. Intracellular organic solutes known as osmolytes are crucial to the integrity of tubular cells within the hypertonic medulla, enabling them to maintain concentrating mechanisms (Fig. 1C).

AVP has a number of other receptors and actions at high concentrations which may have evolved as part of a fight- or flight-type response. Vasopressin Type 1a receptors (V1aR) cause vasoconstriction via smooth muscle cells [16], induce natriuresis [17], and increase renin secretion [14]. AVP V1b receptors can stimulate adrenocorticotropic hormone (ACTH) and therefore stimulate cortisol release [18]. There has been substantial recent interest in AVP, its relationship to metabolic and vascular disease, and whether increased water intake could be of benefit [19].

## Definitions and assessment of dehydration

To understand published studies of hydration, it is important to review how dehydration can be defined and quantified, including the method for this.

The total body water (TBW) state of an individual lies in a spectrum between *hypohydration (dehydration),* euvolaemia, and fluid overload. Dehydration is classically defined as a process of body water loss independent of TBW state, with hypohydration, the state of water deficit. In medicine however, the term dehydration is commonly used in place of hypohydration, and we have therefore elected to use this throughout this paper.

There can be challenges in assessing hydration state because homeostasis means it can be in constant flux [20]. For example, the spectrum between dehydration and euvolaemia is *underhydration*. This is a state of compensated dehydration with homeostasis having restored TBW to euvolaemia [21]. There are also challenges in identifying mildly deranged volume state. Mild dehydration with < 3% TBW deficit is usually undetected by many standard clinical tools, and in this paper we label it as *subclinical dehydration*.

Dehydration generally only produces the classic clinical signs such as reduced skin turgor or tachycardia with loss of 5% or more of body weight [22]. However, even at or above this range, these signs have limited accuracy in assessment of the actual degree of dehydration [23] and there can be challenges in assessment where body compartment tonicity and typical water body distribution are altered, such as in hypernatremia or hypoalbuminemia. Addition of lab tests including standard electrolyte panels with urea, serum creatinine, bicarbonate, and haematocrit can be helpful to detect dehydration [22, 23]. There are however clear drawbacks in CKD with the interpretation of these parameters, and sensitivity for identifying less significant dehydration or underhydration in the broad CKD population is unstudied to our knowledge.

Weight change is perhaps the most useful clinical tool to detect and sensitively quantify acute dehydration [24], but for accuracy, this requires that a prior recent weight at euvolaemia is known, the weight is taken after a void, and the same set of scales are used. Even the most rigorous clinical researcher or nephrologist will face challenges with this. Monitoring of fluid balance via measurement of intake and loss can be a useful tool to complement hydration assessment in the monitored hospital environment, and these parameters have also been used in population research, including to inform adequate fluid intake recommendations, despite inherent inaccuracies [25]. In the healthy population, decrease of urinary water loss is the main homeostatic response to inadequate hydration. Measurement of urine volume alone in this population, especially if there are minimum other insensible losses, can therefore provide information about hydration state and water intake, although accurate quantification can be limited [26].

As the key regulator of hydration, urine concentration indices (urine specific gravity (SG) or urine osmolality (UOsm)) can be helpful in detection of both dehydration and underhydration. Urine colour can correlate with these indices and colour charts have been used in public health messaging to encourage hydration during heat waves. Urine osmolality from sampling over 24 h has better correlation with fluid intake in healthy sedentary adults than first morning urine samples; however, margin of error and sample collection issues limit clinical utility [26]. Recently, in healthy children, late afternoon spot urine osmolality has been shown to have good equivalence with 24-h osmolality with a mean difference of only 62 mmol kg^−1^ [27]. It is important to appreciate that urine concentration indices and volume reflect the state of homeostasis and not necessarily TBW at that moment in time. Thus, in underhydration, these indices are elevated with normal plasma osmolality. This is differentiated from dehydration where plasma osmolality can increase with as little as 1% body weight loss [28]. In the CKD population urinary concentration measures, colour and volumes may not be reliable methods to detect dehydration or underhydration due to impaired kidney concentrating capacity [29], and there are issues with plasma osmolality in CKD as it also reflects the solute urea and other osmoles which are often higher in CKD.

Gold standard quantification of hydration state is generally considered as TBW measured using isotope dilution compared to references; however, this is not a practical clinical tool due to the time, cost, and risks with these studies, which are not able to repeated frequently. It is also unclear exactly where dehydration should be defined based on TBW percentile distribution references [20]. An alternative for TBW assessment is bioelectrical impedance analysis (BIA) which assesses TBW based on the resistance of electric currents within tissues, which changes according to water content. Further research is required to enable routine clinical implementation of BIA in children with CKD, especially for determination of dehydrated states [30]. In non-CKD, point of care ultrasound to measure inferior vena cava (IVC) and aortic (Ao) dimensions has been proposed (IVC/Ao ratio and IVC collapsibility index) to help diagnose and quantify acute dehydration; however, the diagnostic performance of these tools is limited based on current research [31].

Despite limitations of these tools in CKD and need for further study of their utility to detect mild dehydration or underhydration [32], they are the only tools available in clinical settings. We have thus incorporated these into a framework for assessing dehydration risk in CKD (“Hydration recommendations in paediatric CKD” section and Table 1 and 2). In the future, mild dehydration and underhydration may be detected by other evidence of activation of homeostatic mechanisms. Adults with lower habitual fluid intake have higher AVP levels compared to those who consume more fluids, despite similar plasma osmolality [33]. AVP measurement however is problematic as it is highly bound to platelets and is unstable in plasma [34]. Copeptin, which is the C terminal fragment of the AVP prohormone, is released in a 1:1 ratio with secreted AVP, and is considered a surrogate AVP marker [34]. Copeptin in large-scale community adult populations is directly associated with urine osmolality and inversely associated with 24-h urine output [35]. Interpretation of copeptin in CKD must be undertaken with care, as there is a decrease of copeptin clearance with declining GFR [36], necessitating GFR-based correction [37], especially at GFR < 28 [38]. Other than in central diabetes insipidus (DI), there is very limited study of copeptin and its relationship with dehydration or CKD in children.

**Table 1:**
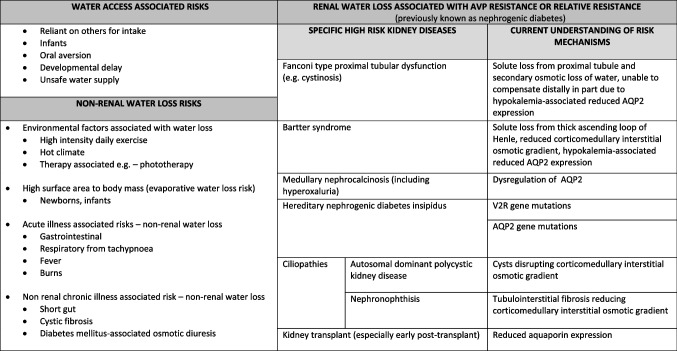
Risk factors for dehydration in childhood CKD

*CKD*, chronic kidney disease; *AQP2*, aquaporin 2 channels; *V2R*, vasopressin type 2 receptor

**Table 2:**
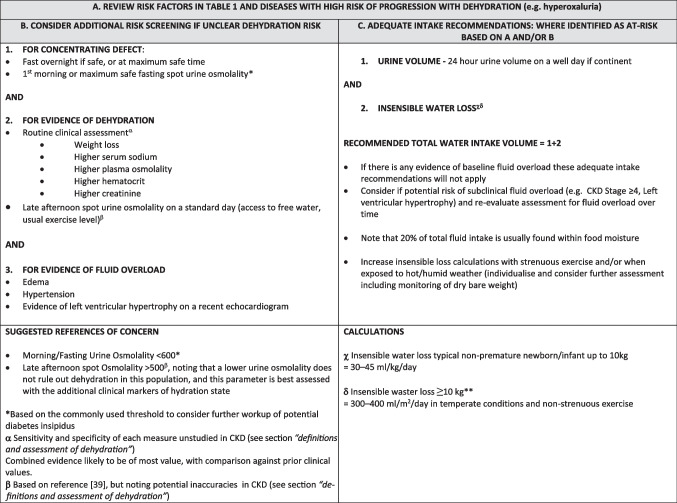
Framework for non-dialysis CKD dehydration risk screening and prevention for outpatient settings

There are no widely accepted specific thresholds of hydration state biomarkers for defining dehydration or underhydration, although some authors have proposed these [39, 40]. A 24-h UOsm of 830 mOsm/kg has been proposed as a threshold definition of dehydration in healthy children and young adults consuming a Western-type diet [40]. Based on studies of health outcomes in adults, including long-term CKD outcomes in the general adult population, a target 24-h UOsm of under 500 mOsm/kg has been quoted to define optimal hydration state [39]. A plasma osmolality level of > 290 mOsm/kg has been used in many studies to define dehydration, with a similar range used as reference to generate North American adequate water intake recommendations [41, 42]. Specific copeptin levels associated with dehydration have not been proposed, although an AVP threshold of ≥ 2 pg/ml has been used in research to define dehydration [43]. There are no biomarkers that reflect a state of chronic dehydration or underhydration. This needs to be taken into account when assessing evidence for association with CKD progression.

## Dehydration risk factors and consequences in CKD

### Risk factors

Risk factors can be grouped into water access associated, and water loss associated, including water loss due to impaired kidney concentrating capacity (Table 1).

Specific hydration challenges in paediatric CKD include the water access dependence of young infants. In the setting of CKD-associated reduced kidney concentrating capacity, patients with reduced water access may have heightened dehydration risk, including acute dehydration associated with other illnesses impacting intake or non-renal water loss. Even with sufficient access to water, inadequate hydration can be common in childhood. In the USA, 54% of healthy 6–19-year-olds were assessed to have inadequate hydration, defined using a UOsm cutoff of > 800 mOsm/kg [44]. Whether there are similar issues for children with CKD is not known.

Impaired concentrating capacity may be the most common predisposing factor for dehydration in CKD. Where defined by fasting urine concentration ≤ 600 mOsm/l, impaired concentrating capacity has been identified in 46% of adult patients with stage 2 CKD, 69% in 3a, and 84% in 3b [29]. There are no similar studies in children to our knowledge. Reduced concentration is attributed to impaired responses to aldosterone and cyclic AMP and AQP to AVP [45, 46]. As CKD progresses, there will be a point where patients are more likely to have baseline fluid overload due to impaired GFR and salt retention. In adults, 20% of outpatients in stage 3–5 CKD (mean eGFR 28.7 ml/min/1.73 m^2)^ have been suggested to have subclinical overhydration based on bioimpedance [47]. In the setting of fluid losses or illness however, many of these patients will be unable to further concentrate their urine and so can still be at higher risk of dehydration.

Aetiology of CKD can be an important risk factor for impaired concentrating capacity. In some cases, this may also be associated with significant polyuria such as in hereditary nephrogenic diabetes insipidus (NDI) and Bartter syndrome. Table 1 presents a list of CKD aetiologies with reported impaired concentrating capacity and the mechanisms responsible for this.

Studies of kidney concentrating capacity and markers of hydration state or acute dehydration episodes in childhood CKD are very limited [48–50]. Even in the more severe cases of AVP resistance, there are few reports in the literature of the consequences of polyuria for these patients. One multicentre cohort study of inherited NDI reported hospitalisation with hypernatremic dehydration subsequent to diagnosis in approximately 28%, and an outcome of CKD stage 2 or more in 30% by median age of 6 years old [51]. It is not clear whether dehydration in these conditions contributes to CKD progression and there are additional risk factors for CKD such as long-term use of COX inhibitors.

In autosomal dominant polycystic kidney disease (ADPKD), reduced concentrating capacity can occur even with high eGFR. In a study of children with mean GFR of > 100 ml/min/1.73 m^2^ undergoing water deprivation, maximal urine concentration was suppressed to a mean of 872 mOsm/kg in ADPKD versus 1041 in healthy controls [48]. In adults with ADPKD this is associated with significantly higher AVP and copeptin [52]. AVP has been shown to be a contributing factor to cyst development due to V2R receptor-triggered increase in cyclic adenosine monophosphate (cAMP) [53, 54], and it is speculated that the cysts themselves also contribute to concentration deficits [55]. Thus, there is theoretical higher risk of dehydration and underhydration in this condition, and via AVP, this may also affect concentrating capacity and disease progression. The pathogenic role of AVP in ADPKD was confirmed with the finding that V2R antagonists (V2RA) were able to reduce renal cAMP and disease progression in animal models [56] and then in ADPKD human disease [57]. The V2RA tolvaptan is now in clinical use for adults with ADPKD who are at high risk of CKD progression. Side effects of therapy, including significant polyuria, limit adherence [57]. Whether reduction in AVP through better hydration can also impact ADPKD progression is unknown. An increase in water intake that was already above standard population levels did not change GFR over 3 years in an Australian adult ADPKD trial [58]. Challenges in this trial meant that water intake did not meet aims, and copeptin levels did not drop; thus, the authors could not definitively conclude that higher water intake is not beneficial to disease progression.

Other diseases with similar ciliary apparatus dysfunction influencing ADH-cAMP-AQP2, with potential dehydration risk, include autosomal recessive polycystic disease (ARPKD) and nephronophthisis (NPH). In ARPKD, renal concentrating defects and osmoregulatory responses have not been well described. In NPH, polyuria and poor urinary concentration is characteristic, and this usually occurs before the decline in GFR. Most recently in Joubert syndrome, which is a syndromic NPH characterised by ciliary defects leading to cerebellar hypoplasia and nephronophthisis in approximately ¼, reduced first morning UOsm with a poor response to desmopressin (a synthetic V2R agonist) has been shown to be associated with CKD progression [59]. Whether poor urinary concentration contributes causally to this has not been investigated.

The concentrated urine of dehydration and underhydration is likely to be an important risk state for progression of nephrocalcinosis and CKD associated with diseases such as hyperoxaluria. The tubular damage of these conditions may also reduce concentrating capacity and exaggerate risk of dehydration. Hyperhydration is an important part of management, increasing the solubility of the compound, theoretically helping to reduce crystallisation and secondary renal damage. There are however no studies to our knowledge that have specifically evaluated the benefits of hyperhydration.

We have elected to exclude from this review lengthy discussion regarding nephrotic syndrome, a state associated with baseline isolated intravascular volume deficit. Readers however should recognise that investigations, physiology, and postulates regarding risk of CKD progression may also apply to chronic nephrotic states. In addition, where acute illness or excessive diuretic use leads to dehydration, this can be more difficult to recognise and there can be a high risk of complications such as acute kidney injury (AKI) and renal vein thrombosis [60].

Hydration in paediatric transplantation can be a challenge. Early post-transplant concentrating capacity may be impaired due to ischaemia reperfusion injury [61] and at this time patients are usually generously hydrated to ensure graft perfusion. By discharge, fluid targets are usually prescribed, but these are often not met [62], and readmission for evaluation of graft dysfunction ultimately attributed to poor intake can be common [50]. Dehydration admissions in the context of infective illness post-transplant are often associated with AKI, and interestingly, Le Page et al. also found that a gastrostomy tube was a risk rather than a protective factor in this context [50]. Formal study of kidney concentrating capacity early post-transplant is limited; however, impaired response to desmopressin was demonstrated by Qvist et al. in the majority of children 1.5 years post-transplant, where mean GFR was between 67 and 85 ml/min/1.73 m^2^, dependent on type of donor kidney [49]. If kidney concentrating capacity is impaired post-transplant, and it is known that fluid targets are often not met, then many patients post-transplant may have chronic dehydration or underhydration. This may be of concern, as in an adult cohort the highest copeptin tertile 6 years post-transplant was shown to associate with a significantly higher subsequent decline in eGFR over 3 years (− 1 ml/min/1.73 m^2^/year versus − 0.03 for the lowest tertile) [63]. Further research is needed to help understand concentrating capacity post-transplant, to help clarify optimal fluid intake recommendations.

### Dehydration and CKD progression — pathophysiologic evidence

Where dehydration is severe and associated with hypovolaemia, renal ischaemia may develop with secondary acute tubular necrosis and kidney injury [64]. Whilst this state is often recoverable, subtle changes in inflammatory pathways can lead to interstitial fibrosis, with potential heightened risk of dehydration due to exacerbated renal concentrating defects, lowering the threshold for further kidney injury and risk of CKD or CKD progression [65]. In more mild dehydration or underhydration where renal blood flow is less disturbed, there is evidence that AVP can drive pathologic processes including via activation of the renin–angiotensin–aldosterone system (RAAS).

In rats, AVP infusion leads in the short term to increased UOsm, increased GFR [66], and albuminuria [67]. In 5/6 nephrectomy rats, those with genetic AVP deficiency had less renal hypertrophy and CKD progression [68]. As reviewed by Meijer et al., use of both V2Ra and/or Vasopressin Type 1a antagonists (V1aRa) in a variety of different rat CKD models reduced or prevented adverse renal outcomes and hypertension in 10 of 11 studies [55]. Mechanisms that may cause AVP-associated hyperfiltration include V2R-mediated sodium resorption at the cortical TAL which alters intraluminal osmolality with secondary changes to macula densa-induced tubulo-glomerular feedback [5]. AVP action at V1aR may contribute to hyperfiltration via increased renin secretion from the juxta-glomerular apparatus [14]. Chronic desmopressin infusion-associated albuminuria in rats can be improved with angiotensin-converting enzyme (ACE) inhibitors [67]. Although GFR can be increased with AVP, glomerular tubular balance and distal AVP actions mean that urine can still be concentrated.

Human studies have confirmed a relationship between underhydration and increased GFR in adults in association with high protein intake [69], and desmopressin infusion has been shown to cause albuminuria, and to increase renin [67]. Studies of GFR in association with desmopressin, however, are limited in humans other than when this is used in critical care where GFR has been shown to increase [67, 70]. The albuminuria associated with desmopressin infusion is V2R dependent, as adult patients with NDI with AQP2 mutations developed desmopressin-associated increased albuminuria, but this did not occur for patients with V2R mutations [67]. It is not known whether RAAS inhibition in humans can change intrarenal haemodynamics or modify CKD progression in response to vasopressin or dehydration.

At a cellular level in rats, AVP has been shown to induce changes that could lead to CKD, including mesangial cell proliferation and TGF-beta-associated collagen production, which can be inhibited by V1AR antagonists (V1ARA) [71, 72].

It also has been proposed that AVP may have a pathogenic role in hypertension [73], theoretically further contributing to CKD progression. Mechanisms however are potentially conflicting. Whilst AVP stimulated V1AR can vasoconstrict [16] and increase renin [14], it can also lead to natriuresis [17]. AVP activation of V2R however can increase sodium resorption (Fig. 1A) [6]. Additional mechanisms that may contribute to hypertension in chronic dehydration include high-osmolality stimulation of CNS sympathetic responses [15] and TonEBP stimulation of serum glucocorticoid-inducible kinase 1 (SGK1) with, again, associated sodium retention [19]. Further study is required to understand if mild dehydration can augment local intrarenal sympathetic nerve activity that can accelerate CKD progression [74].

CKD progression has been predicted by increased urinary osmolytes, which may relate to defective gene expression of associated tubule transporters [75]. As discussed in the “Physiology of water homeostasis” section, these osmolytes protect tubular cells in the high-osmolality renal medulla (Fig. 1C) and their loss may make tubular cells more vulnerable when under the hyperosmotic stress of dehydration. This may then contribute to both worse concentrating capacity and CKD progression.

Another pathway that could contribute to dehydration risks for CKD patients is the polyol pathway. In mice with recurrent heat-induced dehydration, delayed rehydration has been associated with increased creatinine, and inflammatory and fibrotic changes on biopsy [76]. An associated increase in renal cortex sorbitol is seen, which is a consequence of polyol pathway activation, possibly triggered by TonEBP [9]. Sorbitol is an osmolyte that helps maintain tubular cell integrity (Fig. 1C). Sorbitol production, however, comes with a consequence particularly at the level of the proximal tubule where it is converted to fructose, and then further metabolised generating reactive oxygen species, and proinflammatory cytokines [10]. These consequences are exacerbated in rodents when rehydrated with sweetened beverages [77]. There are no human studies to our knowledge that have assessed markers of tubular polyol pathway induction in dehydration; however, in exercise in heat, soft drink consumption versus water intake is associated with mild AKI with increased urine tubular injury biomarkers and copeptin [78].

### Dehydration and CKD progression — clinical evidence

Whilst AKI is a risk factor for CKD [79] or progression of established CKD [80], clinical studies following up outcomes specifically of acute dehydration-associated AKI are lacking. Subacute and recurrent dehydration, however, has been linked to CKD, with dehydration associated with heat exposure hypothesised to be a contributing factor to an epidemic of CKD of unknown aetiology (CKDu) [81]. This disease occurs primarily in young agricultural workers who work in hot conditions, and is characterised by abnormal eGFR and mild proteinuria usually in the absence of acute presentation with AKI [81]. Kidney biopsies demonstrate tubulointerstitial injury, secondary glomerulosclerosis, and signs of glomerular ischaemia [82]. Heat and dehydration, along with use of non-steroidal anti-inflammatory drugs and possibly other toxins, are proposed as primary risk factors for developing CKDu but causality has not been established [81, 83]. Studies of workers at risk demonstrate higher tubular injury biomarkers and raised copeptin [84, 85].

With respect to underhydration or subclinical dehydration, cross-sectional studies of healthy adult populations demonstrate associations between higher UOsm and higher GFR that can extend into the hyperfiltration range [86]. The pathophysiologic studies suggest that AVP may mediate this relationship [67, 69], and adult healthy population studies identify associations of low urine output and high copeptin, with microalbuminuria and increased future risk of CKD [87, 88]. Study of whether ACE inhibition can modify this association would be of interest. One study of healthy children demonstrated that higher morning urine specific gravity was associated with higher urinary microalbumin, although relevance of this finding to dehydration is unclear, as this should not be defined by fasting morning urine [89].

In CKD there are inconsistent findings regarding the association of UOsm and urine volume with CKD progression (Table 3). These studies are all in adult patients and although adjusted for baseline factors, they are difficult to compare, as populations are very different with a range of initial baseline eGFR and different proportions of CKD aetiologies, both of which can influence kidney concentrating capacity [90, 91]. Populations also have variable comorbidities and diuretic use. Assessment of copeptin as a marker of acute hydration state in CKD may have an advantage over UOsm or urine volume-based assessment (Table 3). Longitudinal studies in adults in ADPKD show utility of high copeptin for predicting a better response to tolvaptan [92]. In transplantation, IgA nephropathy and diabetes mellitus, higher baseline copeptin has been associated with a greater decline in GFR over time [63, 90].

**Table 3:**
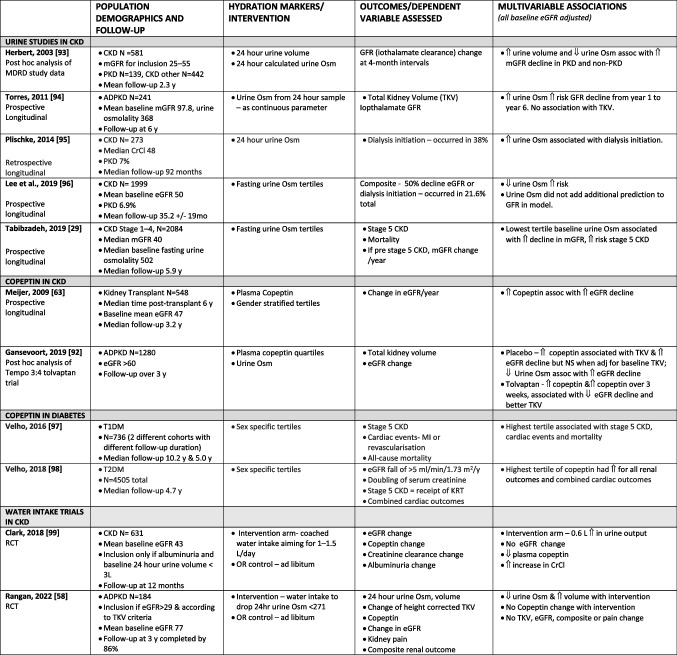
Clinical research of hydration state and CKD outcomes (all adult cohorts with threshold for inclusion *N* > 100)

*CKD*, chronic kidney disease; *N*, number; *L*, litres; *ml*, millilitres; *eGFR*, estimated GFR; *mGFR*, measured GFR; *Osm*, osmolality; *pa*, per annum; *KDIGO*, Kidney Disease Improving Global Outcomes guidelines; *SD*, standard deviation; *NFI*, normal fluid intake; *HFI*, high fluid intake; *ADPKD*, autosomal dominant polycystic kidney disease; *CrCl*, creatinine clearance; *y*, years; *mo*, months; *assoc*, associated with; *IFTA*, interstitial fibrosis and tubular atrophy; *omso*, osmolality; *TKV*, total kidney volume; *T1DM*, type 1 diabetes mellitus; *T2DM*, type 2 diabetes mellitus; *CV*, cardiovascular; *KRT*, kidney replacement therapy; *RCT*, randomised clinical trial

Water intake studies in adult CKD have not shown a clear-cut association of improved hydration with limiting GFR decline. In a randomised trial of coached water intake in CKD stage 3 assessing a primary outcome of eGFR change over 12 months, for an average 0.7-L increase in water intake for the intervention group, copeptin decreased, but no significant eGFR change was seen [99]. This study had limitations and the number of enrolled participants did not meet the sample size goal. The previously described water intake trial in ADPKD with a mean eGFR of 77 ml/min/1.73 m^2^ also did not show a benefit of improved hydration with eGFR change over 3 years [58]. There are significant methodologic challenges in these trials, including compliance with the intervention. If AVP is an important pathophysiologic factor in CKD progression, and water intake supresses AVP, then evaluation of subcohorts who have more significant AVP or copeptin decline is important to establish whether better hydration can reduce CKD progression.

Most of the studies referenced in Table 3 are for populations with mean age above 40. In children, studies of hydration and association with CKD are limited. One recent study demonstrated that plasma copeptin was higher in young adults born preterm versus controls, and within the preterm group, copeptin was higher in those with more severe neonatal course, smaller kidney volume, and albuminuria [100].

If AVP is a significant contributor to progressive kidney disease, then it would be expected that chronic SIADH (syndrome of inappropriate antidiuretic hormone) or overuse of desmopressin would lead to CKD. Similarly, one would expect that nephrogenic syndrome of inappropriate antidiuresis (NSIAD), which is due to gain of function mutations of V2R, would also associate with CKD. The only relevant study to our knowledge is a report on a small mixed cohort of adults with SIADH, desmopressin overuse, and NSIAD, which did not show an association with a single 24-h measure of microalbuminuria [101].

From the adult studies, can we conclude that subclinical dehydration or underhydration, especially where chronic, could contribute to CKD progression, including in children? The key evidence for the pro-argument is the association of copeptin with CKD progression in the epidemiologic studies, which is supported by pathophysiology. However, it needs to be acknowledged that there may be publication bias and confounding, and that rodent physiology and human AVP infusion studies do not represent everyday human physiology. In addition, the studies referenced examine copeptin levels at a single time point, which does not identify a chronic state of dehydration or underhydration. For the con-argument, are the hydration trials. These trials, however, did not specifically evaluate cohorts at highest risk of dehydration, and it is possible that the effects of dehydration may only be borne out over the longer term. A definitive answer to this question may be difficult to establish. Further clinical research into CKD populations with a high vulnerability to chronic states of dehydration and underhydration, and CKD populations with fewer comorbidities, such as children, may be helpful.

### Other health consequences of subclinical dehydration and underhydration

Epidemiologic studies in adults have associated copeptin as a marker of subclinical dehydration and underhydration, with metabolic dysfunction including diabetes and obesity [88]. Additionally, there is some evidence suggesting that dehydration can impact cardiovascular health and inflammation [102]. Ex vivo and rodent studies show that high plasma osmolality induces endothelial expression of pro-inflammatory mediators, with at-risk mice undergoing water restriction demonstrating more advanced atherosclerotic lesions [103]. In a cross-over study in healthy men, mild dehydration in association with exercise and fluid restriction has been shown to increase markers of vascular stiffness [104]. Mediators of this response were not investigated, although it was proposed that RAAS activation and secondary changes in endothelial nitric oxide were implicated. Assuming that the pathophysiologic mechanisms that underlie these associations are shared by both adults and children, this may be of relevance over a lifetime for those with childhood CKD with chronic or recurrent subclinical dehydration and underhydration, who already have higher baseline risk of cardiovascular disease.

An acute impact that has been associated with mild dehydration or underhydration in children is cognitive dysfunction [105], and this has been shown to improve with water intake [106]. Study of associated hydration biomarkers and cognitive function would be of interest in the well CKD population.

## Hydration recommendations in paediatric CKD

Although there is need for further research into the consequences of dehydration and underhydration in the CKD population, as paediatric nephrology clinicians we should still have a precautionary approach and provide balanced guidance on adequate water intake, especially for higher risk patients.

For healthy children, adequate total water intake recommendations have been published by European and North American nutritional authorities [42, 107] (Table 4). The North American recommendations were based on median general population total fluid intake from a surveyed population with no significant dehydration based on a plasma osmolality definition. The European recommendations were also based on childhood intake studies but incorporated desirable urine concentration (UOsm < 500 mOsm/l) into the recommendations. The adequate intake recommendations have limitations, including derivation from intake studies of broad populations with substantially different water loss factors [25]. A recent global study of water turnover using isotope tracking methods may more accurately inform water intake recommendations including under different environmental conditions and according to physical activity [108].

**Table 4:** Healthy children adequate total water intake recommendations (litres) — derived from [25]

	European [107]		North American [42]	
Age	Total water intake	Fluid intake (80% total water intake)	Total water intake	Fluid intake (beverage intake)
0–6 months	0.68	0.68	0.70	0.70
6–12 months	0.80–1.0	0.64–0.80		
7–12 months			0.80	0.80
1–2 years	1.1–1.2	0.88–0.90		
2–3 years	1.3	1.0		
1–3 years			1.3	0.9
4–8 years	1.6	1.2	1.7	1.2
9–13 years				
Boys	2.1	1.6	2.4	1.8
Girls	1.9	1.5	2.1	1.6
> 14 years				
Boys	2.5	2	3.3	2.6
Girls	2.0	1.6	2.3	1.8

For children with CKD, these water intake recommendations do not take into account impaired kidney concentrating ability or risk of baseline fluid overload. Thus, an individual assessment of hydration state and dehydration risk should be considered (Table 1 and 2). Thirst-guided water intake, with additional intake in hot environments or with physical activity, may be the right guidance for most well paediatric CKD patients who do not clearly fall into the extremes of hydration risk states. We acknowledge that there can be subclinical fluid overload in moderate to severe CKD; however, where salt is adequately restricted, again, thirst-guided water intake is usually appropriate with caveats about sick days especially with vomiting or diarrhoea. Further translational research of markers that may identify subclinical dehydration, underhydration, and fluid overload, such as bioimpedance, may help clarify optimal guidance. Based on current knowledge, however, screening at-risk CKD patients for impaired urine concentration and evidence of inadequate hydration, with expert interpretation of hydration state and provision of balanced intake advice, could be considered as per Table 2. Initial proposed fluid increments should be suggested in extremes of weather, exercise, or illness but re-evaluated according to weight change or other monitored laboratory parameters.

## Summary

Despite potential vulnerability due to factors including age and disease aetiology, there is limited published research on dehydration and relevance in childhood CKD. It is biologically plausible that dehydration and underhydration affect CKD progression, and there may be other health implications of inadequate water intake for children. AVP is potentially a key mediator of these risks, and copeptin, which is an easily measured AVP surrogate that is reduced with water intake, is associated with CKD outcomes in adult cohorts. Trials of increased water intake in CKD and ADPKD in adults have not demonstrated attenuation of CKD progression. Vulnerable patients who have high baseline copeptin may have the most to gain from increased water intake, and trials stratifying therapeutic outcomes of water intake based on baseline copeptin level would be of interest. Future studies are required to clarify how common dehydration and underhydration is for the paediatric CKD population, and its impacts including hospital admissions and evidence for risk of CKD progression. For now, a risk factor-based stratification for providing hydration guidance in paediatric CKD clinics should be considered.

## Supplementary Information

Below is the link to the electronic supplementary material.

Graphical abstract (PPTX 136 KB)

## References

[1] Danziger J, Zeidel ML (2015) Osmotic homeostasis. Clin J Am Soc Nephrol 10:852–862. 10.2215/cjn.10741013 25078421 10.2215/CJN.10741013 PMC4422250

[2] Hughes F, Mythen M, Montgomery H (2018) The sensitivity of the human thirst response to changes in plasma osmolality: a systematic review. Perioper Med (Lond) 7:1. 10.1186/s13741-017-0081-4 29344350 10.1186/s13741-017-0081-4 PMC5763530

[3] Kimura T, Minai K, Matsui K, Mouri T, Sato T (1976) Effect of various states of hydration on plasma ADH and renin in man. J Clin Endocrinol Metab 42:79–87. 10.1210/jcem-42-1-79 1249195 10.1210/jcem-42-1-79

[4] Sands JM, Layton HE (2009) The physiology of urinary concentration: an update. Semin Nephrol 29:178–195. 10.1016/j.semnephrol.2009.03.008 19523568 10.1016/j.semnephrol.2009.03.008 PMC2709207

[5] Bachmann S, Mutig K (2017) Regulation of renal Na-(K)-Cl cotransporters by vasopressin. Pflugers Arch 469:889–897. 10.1007/s00424-017-2002-2 28577072 10.1007/s00424-017-2002-2

[6] Bankir L, Fernandes S, Bardoux P, Bouby N, Bichet DG (2005) Vasopressin-V2 receptor stimulation reduces sodium excretion in healthy humans. J Am Soc Nephrol 16:1920–1928. 10.1681/asn.2004121079 15888562 10.1681/ASN.2004121079

[7] Garcia-Perez A, Burg MB (1991) Role of organic osmolytes in adaptation of renal cells to high osmolality. J Membr Biol 119:1–13. 10.1007/bf01868535 1901090 10.1007/BF01868535

[8] Hasler U, Jeon US, Kim JA, Mordasini D, Kwon HM, Féraille E, Martin PY (2006) Tonicity-responsive enhancer binding protein is an essential regulator of aquaporin-2 expression in renal collecting duct principal cells. J Am Soc Nephrol 17:1521–1531. 10.1681/asn.2005121317 16641150 10.1681/ASN.2005121317

[9] Ko BC, Ruepp B, Bohren KM, Gabbay KH, Chung SS (1997) Identification and characterization of multiple osmotic response sequences in the human aldose reductase gene. J Biol Chem 272:16431–16437. 10.1074/jbc.272.26.16431 9195951 10.1074/jbc.272.26.16431

[10] Nakagawa T, Johnson RJ, Andres-Hernando A, Roncal-Jimenez C, Sanchez-Lozada LG, Tolan DR, Lanaspa MA (2020) Fructose production and metabolism in the kidney. J Am Soc Nephrol 31:898–906. 10.1681/asn.2019101015 32253274 10.1681/ASN.2019101015 PMC7217403

[11] Wang Y, Klein JD, Blount MA, Martin CF, Kent KJ, Pech V, Wall SM, Sands JM (2009) Epac regulates UT-A1 to increase urea transport in inner medullary collecting ducts. J Am Soc Nephrol 20:2018–2024. 10.1681/asn.2008121225 19661162 10.1681/ASN.2008121225 PMC2736771

[12] Nakayama Y, Peng T, Sands JM, Bagnasco SM (2000) The TonE/TonEBP pathway mediates tonicity-responsive regulation of UT-A urea transporter expression. J Biol Chem 275:38275–38280. 10.1074/jbc.M004678200 10995747 10.1074/jbc.M004678200

[13] Chapman CL, Johnson BD, Parker MD, Hostler D, Pryor RR, Schlader Z (2021) Kidney physiology and pathophysiology during heat stress and the modification by exercise, dehydration, heat acclimation and aging. Temperature (Austin) 8:108–159. 10.1080/23328940.2020.1826841 33997113 10.1080/23328940.2020.1826841 PMC8098077

[14] Aoyagi T, Izumi Y, Hiroyama M, Matsuzaki T, Yasuoka Y, Sanbe A, Miyazaki H, Fujiwara Y, Nakayama Y, Kohda Y, Yamauchi J, Inoue T, Kawahara K, Saito H, Tomita K, Nonoguchi H, Tanoue A (2008) Vasopressin regulates the renin-angiotensin-aldosterone system via V1a receptors in macula densa cells. Am J Physiol Renal Physiol 295:F100-107. 10.1152/ajprenal.00088.2008 18448596 10.1152/ajprenal.00088.2008

[15] Toney GM, Stocker SD (2010) Hyperosmotic activation of CNS sympathetic drive: implications for cardiovascular disease. J Physiol 588:3375–3384. 10.1113/jphysiol.2010.191940 20603334 10.1113/jphysiol.2010.191940 PMC2988504

[16] Henderson KK (1985) Byron KL (2007) Vasopressin-induced vasoconstriction: two concentration-dependent signaling pathways. J Appl Physiol 102:1402–1409. 10.1152/japplphysiol.00825.2006 10.1152/japplphysiol.00825.2006 PMC2580829 17204577

[17] Perucca J, Bichet DG, Bardoux P, Bouby N, Bankir L (2008) Sodium excretion in response to vasopressin and selective vasopressin receptor antagonists. J Am Soc Nephrol 19:1721–1731. 10.1681/asn.2008010021 18596120 10.1681/ASN.2008010021 PMC2518442

[18] Tanoue A, Ito S, Honda K, Oshikawa S, Kitagawa Y, Koshimizu TA, Mori T, Tsujimoto G (2004) The vasopressin V1b receptor critically regulates hypothalamic-pituitary-adrenal axis activity under both stress and resting conditions. J Clin Invest 113:302–309. 10.1172/jci19656 14722621 10.1172/JCI19656 PMC311433

[19] Lang F, Guelinckx I, Lemetais G, Melander O (2017) Two liters a day keep the doctor away? Considerations on the pathophysiology of suboptimal fluid intake in the common population. Kidney Blood Press Res 42:483–494. 10.1159/000479640 28787716 10.1159/000479640

[20] Armstrong LE (2007) Assessing hydration status: the elusive gold standard. J Am Coll Nutr 26:575s–584s. 10.1080/07315724.2007.10719661 17921468 10.1080/07315724.2007.10719661

[21] Kavouras SA (2019) Hydration, dehydration, underhydration, optimal hydration: are we barking up the wrong tree? Eur J Nutr 58:471–473. 10.1007/s00394-018-01889-z 30607564 10.1007/s00394-018-01889-z

[22] Vega RM, Avner JR (1997) A prospective study of the usefulness of clinical and laboratory parameters for predicting percentage of dehydration in children. Pediatr Emerg Care 13:179–182. 10.1097/00006565-199706000-00001 9220501 10.1097/00006565-199706000-00001

[23] Ugale J, Mata A, Meert KL, Sarnaik AP (2012) Measured degree of dehydration in children and adolescents with type 1 diabetic ketoacidosis. Pediatr Crit Care Med 13:e103-107. 10.1097/PCC.0b013e3182231493 21666534 10.1097/PCC.0b013e3182231493

[24] Bruck E (1971) Laboratory tests in the analysis of states of dehydration. Pediatr Clin North Am 18:265–283. 10.1016/s0031-3955(16)32538-x 25868190 10.1016/s0031-3955(16)32538-x

[25] Gandy J (2015) Water intake: validity of population assessment and recommendations. Eur J Nutr 54(Suppl 2):11–16. 10.1007/s00394-015-0944-8 26048039 10.1007/s00394-015-0944-8 PMC4473081

[26] Perrier E, Rondeau P, Poupin M, Le Bellego L, Armstrong LE, Lang F, Stookey J, Tack I, Vergne S, Klein A (2013) Relation between urinary hydration biomarkers and total fluid intake in healthy adults. Eur J Clin Nutr 67:939–943. 10.1038/ejcn.2013.93 23695204 10.1038/ejcn.2013.93 PMC3778844

[27] Suh H, Summers LG, Seal AD, Colburn AT, Mauromoustakos A, Perrier ET, Bottin JH, Kavouras SA (2020) Afternoon urine osmolality is equivalent to 24 h for hydration assessment in healthy children. Eur J Clin Nutr 74:884–890. 10.1038/s41430-019-0519-5 31624367 10.1038/s41430-019-0519-5 PMC7283040

[28] Popowski LA, Oppliger RA, Patrick Lambert G, Johnson RF, Kim Johnson A, Gisolf CV (2001) Blood and urinary measures of hydration status during progressive acute dehydration. Med Sci Sports Exerc 33:747–753. 10.1097/00005768-200105000-00011 11323543 10.1097/00005768-200105000-00011

[29] Tabibzadeh N, Wagner S, Metzger M, Flamant M, Houillier P, Boffa JJ, Vrtovsnik F, Thervet E, Stengel B, Haymann JP (2019) Fasting urinary osmolality, CKD progression, and mortality: a prospective observational study. Am J Kidney Dis 73:596–604. 10.1053/j.ajkd.2018.12.024 30777634 10.1053/j.ajkd.2018.12.024

[30] Dasgupta I, Keane D, Lindley E, Shaheen I, Tyerman K, Schaefer F, Wühl E, Müller MJ, Bosy-Westphal A, Fors H, Dahlgren J, Chamney P, Wabel P, Moissl U (2018) Validating the use of bioimpedance spectroscopy for assessment of fluid status in children. Pediatr Nephrol 33:1601–1607. 10.1007/s00467-018-3971-x 29869117 10.1007/s00467-018-3971-x PMC6061658

[31] Kaminecki I, Huang DM, Shipman PC, Gibson RW (2023) Point-of-care ultrasonography for the assessment of dehydration in children: a systematic review. Pediatr Emerg Care 39:786–796. 10.1097/pec.0000000000003025 37562138 10.1097/PEC.0000000000003025

[32] Kanbay M, Yilmaz S, Dincer N, Ortiz A, Sag AA, Covic A, Sánchez-Lozada LG, Lanaspa MA, Cherney DZI, Johnson RJ, Afsar B (2019) Antidiuretic hormone and serum osmolarity physiology and related outcomes: what is old, what is new, and what is unknown? J Clin Endocrinol Metab 104:5406–5420. 10.1210/jc.2019-01049 31365096 10.1210/jc.2019-01049

[33] Perrier E, Vergne S, Klein A, Poupin M, Rondeau P, Le Bellego L, Armstrong LE, Lang F, Stookey J, Tack I (2013) Hydration biomarkers in free-living adults with different levels of habitual fluid consumption. Br J Nutr 109:1678–1687. 10.1017/s0007114512003601 22935250 10.1017/S0007114512003601 PMC3638312

[34] Balanescu S, Kopp P, Gaskill MB, Morgenthaler NG, Schindler C, Rutishauser J (2011) Correlation of plasma copeptin and vasopressin concentrations in hypo-, iso-, and hyperosmolar states. J Clin Endocrinol Metab 96:1046–1052. 10.1210/jc.2010-2499 21289257 10.1210/jc.2010-2499

[35] Meijer E, Bakker SJ, Halbesma N, de Jong PE, Struck J, Gansevoort RT (2010) Copeptin, a surrogate marker of vasopressin, is associated with microalbuminuria in a large population cohort. Kidney Int 77:29–36. 10.1038/ki.2009.397 19847155 10.1038/ki.2009.397

[36] Benmansour M, Rainfray M, Paillard F, Ardaillou R (1982) Metabolic clearance rate of immunoreactive vasopressin in man. Eur J Clin Invest 12:475–480. 10.1111/j.1365-2362.1982.tb02228.x 6818035 10.1111/j.1365-2362.1982.tb02228.x

[37] Roussel R, Fezeu L, Marre M, Velho G, Fumeron F, Jungers P, Lantieri O, Balkau B, Bouby N, Bankir L, Bichet DG (2014) Comparison between copeptin and vasopressin in a population from the community and in people with chronic kidney disease. J Clin Endocrinol Metab 99:4656–4663. 10.1210/jc.2014-2295 25202818 10.1210/jc.2014-2295

[38] Ettema EM, Heida J, Casteleijn NF, Boesten L, Westerhuis R, Gaillard C, Gansevoort RT, Franssen CFM, Zittema D (2017) The effect of renal function and hemodialysis treatment on plasma vasopressin and copeptin levels. Kidney Int Rep 2:410–419. 10.1016/j.ekir.2017.01.006 29142968 10.1016/j.ekir.2017.01.006 PMC5678637

[39] Perrier ET, Buendia-Jimenez I, Vecchio M, Armstrong LE, Tack I, Klein A (2015) Twenty-four-hour urine osmolality as a physiological index of adequate water intake. Dis Markers 2015:231063. 10.1155/2015/231063 25866433 10.1155/2015/231063 PMC4381985

[40] Manz F, Wentz A (2003) 24-h hydration status: parameters, epidemiology and recommendations. Eur J Clin Nutr 57(Suppl 2):S10-18. 10.1038/sj.ejcn.1601896 14681708 10.1038/sj.ejcn.1601896

[41] Baron S, Courbebaisse M, Lepicard EM, Friedlander G (2015) Assessment of hydration status in a large population. Br J Nutr 113:147–158. 10.1017/s0007114514003213 25418739 10.1017/S0007114514003213

[42] Institute of Medicine FaNB. Dietary reference intakes for water, potassium, sodium, chloride and sulfate. In: National Academies Press W, DC, editor. 2004

[43] Armstrong LE, Johnson EC (2018) Water intake, water balance, and the elusive daily water requirement. Nutrients 10:1928. 10.3390/nu10121928 30563134 10.3390/nu10121928 PMC6315424

[44] Kenney EL, Long MW, Cradock AL, Gortmaker SL (2015) Prevalence of inadequate hydration among US children and disparities by gender and race/ethnicity: National Health and Nutrition Examination Survey, 2009–2012. Am J Public Health 105:e113-118. 10.2105/ajph.2015.302572 26066941 10.2105/AJPH.2015.302572 PMC4504329

[45] Pedersen EB, Thomsen IM, Lauridsen TG (2010) Abnormal function of the vasopressin-cyclic-AMP-aquaporin2 axis during urine concentrating and diluting in patients with reduced renal function. A case control study BMC Nephrol 11:26. 10.1186/1471-2369-11-26 20923561 10.1186/1471-2369-11-26 PMC2965705

[46] Malmberg MH, Mose FH, Pedersen EB, Bech JN (2020) Urine concentration ability is reduced to the same degree in adult dominant polycystic kidney disease compared with other chronic kidney diseases in the same CKD-stage and lower than in healthy control subjects — a case control study. BMC Nephrol 21:379. 10.1186/s12882-020-02043-w 32867720 10.1186/s12882-020-02043-w PMC7457520

[47] Hung SC, Kuo KL, Peng CH, Wu CH, Lien YC, Wang YC, Tarng DC (2014) Volume overload correlates with cardiovascular risk factors in patients with chronic kidney disease. Kidney Int 85:703–709. 10.1038/ki.2013.336 24025647 10.1038/ki.2013.336

[48] Ho TA, Godefroid N, Gruzon D, Haymann JP, Maréchal C, Wang X, Serra A, Pirson Y, Devuyst O (2012) Autosomal dominant polycystic kidney disease is associated with central and nephrogenic defects in osmoregulation. Kidney Int 82:1121–1129. 10.1038/ki.2012.225 22718190 10.1038/ki.2012.225

[49] Qvist E, Laine J, Rönnholm K, Jalanko H, Leijala M, Holmberg C (1999) Graft function 5–7 years after renal transplantation in early childhood. Transplantation 67:1043–1049. 10.1097/00007890-199904150-00018 10221491 10.1097/00007890-199904150-00018

[50] Le Page AK, Johnstone LM, Kausman JY (2023) Hospital admissions associated with dehydration in childhood kidney transplantation. Pediatr Nephrol. 10.1007/s00467-023-06095-6 37555933 10.1007/s00467-023-06095-6 PMC10728223

[51] D’Alessandri-Silva C, Carpenter M, Ayoob R, Barcia J, Chishti A, Constantinescu A, Dell KM, Goodwin J, Hashmat S, Iragorri S, Kaspar C, Mason S, Misurac JM, Muff-Luett M, Sethna C, Shah S, Weng P, Greenbaum LA, Mahan JD (2019) Diagnosis, treatment, and outcomes in children with congenital nephrogenic diabetes insipidus: a pediatric nephrology research consortium study. Front Pediatr 7:550. 10.3389/fped.2019.00550 32039113 10.3389/fped.2019.00550 PMC6985429

[52] Zittema D, Boertien WE, van Beek AP, Dullaart RP, Franssen CF, de Jong PE, Meijer E, Gansevoort RT (2012) Vasopressin, copeptin, and renal concentrating capacity in patients with autosomal dominant polycystic kidney disease without renal impairment. Clin J Am Soc Nephrol 7:906–913. 10.2215/cjn.11311111 22516290 10.2215/CJN.11311111

[53] Janssens P, Weydert C, De Rechter S, Wissing KM, Liebau MC, Mekahli D (2018) Expanding the role of vasopressin antagonism in polycystic kidney diseases: from adults to children? Pediatr Nephrol 33:395–408. 10.1007/s00467-017-3672-x 28455745 10.1007/s00467-017-3672-x

[54] Hanaoka K, Guggino WB (2000) cAMP regulates cell proliferation and cyst formation in autosomal polycystic kidney disease cells. J Am Soc Nephrol 11:1179–1187. 10.1681/asn.V1171179 10864573 10.1681/ASN.V1171179

[55] Meijer E, Boertien WE, Zietse R, Gansevoort RT (2011) Potential deleterious effects of vasopressin in chronic kidney disease and particularly autosomal dominant polycystic kidney disease. Kidney Blood Press Res 34:235–244. 10.1159/000326902 21691126 10.1159/000326902

[56] Torres VE, Wang X, Qian Q, Somlo S, Harris PC, Gattone VH 2nd (2004) Effective treatment of an orthologous model of autosomal dominant polycystic kidney disease. Nat Med 10:363–364. 10.1038/nm1004 14991049 10.1038/nm1004

[57] Torres VE, Chapman AB, Devuyst O, Gansevoort RT, Grantham JJ, Higashihara E, Perrone RD, Krasa HB, Ouyang J, Czerwiec FS (2012) Tolvaptan in patients with autosomal dominant polycystic kidney disease. N Engl J Med 367:2407–2418. 10.1056/NEJMoa1205511 23121377 10.1056/NEJMoa1205511 PMC3760207

[58] Rangan GK, Wong ATY, Munt A, Zhang JQJ, Saravanabavan S, Louw S, Allman-Farinelli M, Badve SV, Boudville N, Chan J, Coolican H, Coulshed S, Edwards ME, Erickson BJ, Fernando M, Foster S, Gregory AV, Haloob I, Hawley CM, Holt J, Howard K, Howell M, Johnson DW, Kline TL, Kumar K, Lee VW, Lonergan M, Mai J, McCloud P, Pascoe E, Peduto A, Rangan A, Roger SD, Sherfan J, Sud K, Torres VE, Vilayur E, Harris DCH (2022) Prescribed water intake in autosomal dominant polycystic kidney disease. NEJM Evid 1:EVIDoa2100021. 10.1056/EVIDoa2100021 38319283 10.1056/EVIDoa2100021

[59] Nuovo S, Fuiano L, Micalizzi A, Battini R, Bertini E, Borgatti R, Caridi G, D’Arrigo S, Fazzi E, Fischetto R, Ghiggeri GM, Giordano L, Leuzzi V, Romaniello R, Signorini S, Stringini G, Zanni G, Romani M, Valente EM, Emma F (2020) Impaired urinary concentration ability is a sensitive predictor of renal disease progression in Joubert syndrome. Nephrol Dial Transplant 35:1195–1202. 10.1093/ndt/gfy333 30403813 10.1093/ndt/gfy333 PMC7417010

[60] Meyrier A, Niaudet P (2018) Acute kidney injury complicating nephrotic syndrome of minimal change disease. Kidney Int 94:861–869. 10.1016/j.kint.2018.04.024 29980292 10.1016/j.kint.2018.04.024

[61] Hussein AA, El-Dken ZH, Barakat N, Abol-Enein H (2012) Renal ischaemia/reperfusion injury: possible role of aquaporins. Acta Physiol (Oxf) 204:308–316. 10.1111/j.1748-1716.2011.02372.x 21992594 10.1111/j.1748-1716.2011.02372.x

[62] Kullgren KA, Scholl P, Kidwell KM, Hmiel SP (2015) Using an interactive water bottle to target fluid adherence in pediatric kidney transplant recipients: a pilot study. Pediatr Transplant 19:35–41. 10.1111/petr.12385 25388882 10.1111/petr.12385

[63] Meijer E, Bakker SJ, de Jong PE, Homan van der Heide JJ, van Son WJ, Struck J, Lems SP, Gansevoort RT (2009) Copeptin, a surrogate marker of vasopressin, is associated with accelerated renal function decline in renal transplant recipients. Transplantation 88:561–567. 10.1097/TP.0b013e3181b11ae4 19696640 10.1097/TP.0b013e3181b11ae4

[64] Ruas AFL, Lébeis GM, de Castro NB, Palmeira VA, Costa LB, Lanza K, Simões e Silva AC (2022) Acute kidney injury in pediatrics: an overview focusing on pathophysiology. Pediatr Nephrol 37:2037–2052. 10.1007/s00467-021-05346-8 34845510 10.1007/s00467-021-05346-8

[65] Parr SK, Siew ED (2016) Delayed consequences of acute kidney injury. Adv Chronic Kidney Dis 23:186–194. 10.1053/j.ackd.2016.01.014 27113695 10.1053/j.ackd.2016.01.014 PMC4849427

[66] Bouby N, Ahloulay M, Nsegbe E, Déchaux M, Schmitt F, Bankir L (1996) Vasopressin increases glomerular filtration rate in conscious rats through its antidiuretic action. J Am Soc Nephrol 7:842–851. 10.1681/asn.V76842 8793792 10.1681/ASN.V76842

[67] Bardoux P, Bichet DG, Martin H, Gallois Y, Marre M, Arthus MF, Lonergan M, Ruel N, Bouby N, Bankir L (2003) Vasopressin increases urinary albumin excretion in rats and humans: involvement of V2 receptors and the renin-angiotensin system. Nephrol Dial Transplant 18:497–506. 10.1093/ndt/18.3.497 12584270 10.1093/ndt/18.3.497

[68] Bouby N, Hassler C, Bankir L (1999) Contribution of vasopressin to progression of chronic renal failure: study in Brattleboro rats. Life Sci 65:991–1004. 10.1016/s0024-3205(99)00330-6 10499867 10.1016/s0024-3205(99)00330-6

[69] Hadj-Aïssa A, Bankir L, Fraysse M, Bichet DG, Laville M, Zech P, Pozet N (1992) Influence of the level of hydration on the renal response to a protein meal. Kidney Int 42:1207–1216. 10.1038/ki.1992.406 1453605 10.1038/ki.1992.406

[70] Bragadottir G, Redfors B, Nygren A, Sellgren J, Ricksten SE (2009) Low-dose vasopressin increases glomerular filtration rate, but impairs renal oxygenation in post-cardiac surgery patients. Acta Anaesthesiol Scand 53:1052–1059. 10.1111/j.1399-6576.2009.02037.x 19572935 10.1111/j.1399-6576.2009.02037.x

[71] Tahara A, Tsukada J, Tomura Y, Suzuki T, Yatsu T, Shibasaki M (2007) Effect of YM218, a nonpeptide vasopressin V(1A) receptor-selective antagonist, on rat mesangial cell hyperplasia and hypertrophy. Vascul Pharmacol 46:463–469. 10.1016/j.vph.2007.02.001 17395547 10.1016/j.vph.2007.02.001

[72] Tahara A, Tsukada J, Tomura Y, Yatsu T, Shibasaki M (2008) Vasopressin increases type IV collagen production through the induction of transforming growth factor-beta secretion in rat mesangial cells. Pharmacol Res 57:142–150. 10.1016/j.phrs.2008.01.003 18299204 10.1016/j.phrs.2008.01.003

[73] Bankir L, Bichet DG, Bouby N (2010) Vasopressin V2 receptors, ENaC, and sodium reabsorption: a risk factor for hypertension? Am J Physiol Renal Physiol 299:F917-928. 10.1152/ajprenal.00413.2010 20826569 10.1152/ajprenal.00413.2010

[74] Kaur J, Young BE, Fadel PJ (2017) Sympathetic overactivity in chronic kidney disease: consequences and mechanisms. Int J Mol Sci 18:1682. 10.3390/ijms18081682 28767097 10.3390/ijms18081682 PMC5578072

[75] Gil RB, Ortiz A, Sanchez-Niño MD, Markoska K, Schepers E, Vanholder R, Glorieux G, Schmitt-Kopplin P, Heinzmann SS (2018) Increased urinary osmolyte excretion indicates chronic kidney disease severity and progression rate. Nephrol Dial Transplant 33:2156–2164. 10.1093/ndt/gfy020 29554320 10.1093/ndt/gfy020

[76] Roncal Jimenez CA, Ishimoto T, Lanaspa MA, Rivard CJ, Nakagawa T, Ejaz AA, Cicerchi C, Inaba S, Le M, Miyazaki M, Glaser J, Correa-Rotter R, González MA, Aragón A, Wesseling C, Sánchez-Lozada LG, Johnson RJ (2014) Fructokinase activity mediates dehydration-induced renal injury. Kidney Int 86:294–302. 10.1038/ki.2013.492 24336030 10.1038/ki.2013.492 PMC4120672

[77] García-Arroyo FE, Tapia E, Muñoz-Jiménez I, Gonzaga-Sánchez G, Arellano-Buendía AS, Osorio-Alonso H, Manterola-Romero L, Roncal-Jiménez CA, Johnson RJ, Sánchez-Lozada LG (2020) Fluid intake restriction concomitant to sweetened beverages hydration induce kidney damage. Oxid Med Cell Longev 2020:8850266. 10.1155/2020/8850266 33354281 10.1155/2020/8850266 PMC7735828

[78] Chapman CL, Johnson BD, Sackett JR, Parker MD, Schlader ZJ (2019) Soft drink consumption during and following exercise in the heat elevates biomarkers of acute kidney injury. Am J Physiol Regul Integr Comp Physiol 316:R189–R198. 10.1152/ajpregu.00351.2018 30601706 10.1152/ajpregu.00351.2018

[79] Greenberg JH, Coca S, Parikh CR (2014) Long-term risk of chronic kidney disease and mortality in children after acute kidney injury: a systematic review. BMC Nephrol 15:184. 10.1186/1471-2369-15-184 25416588 10.1186/1471-2369-15-184 PMC4251927

[80] Hsu RK, Hsu CY (2016) The role of acute kidney injury in chronic kidney disease. Semin Nephrol 36:283–292. 10.1016/j.semnephrol.2016.05.005 27475659 10.1016/j.semnephrol.2016.05.005 PMC4979984

[81] Jayasumana C, Orantes C, Herrera R, Almaguer M, Lopez L, Silva LC, Ordunez P, Siribaddana S, Gunatilake S, De Broe ME (2017) Chronic interstitial nephritis in agricultural communities: a worldwide epidemic with social, occupational and environmental determinants. Nephrol Dial Transplant 32:234–241. 10.1093/ndt/gfw346 28186530 10.1093/ndt/gfw346

[82] Wijkström J, Leiva R, Elinder CG, Leiva S, Trujillo Z, Trujillo L, Söderberg M, Hultenby K, Wernerson A (2013) Clinical and pathological characterization of Mesoamerican nephropathy: a new kidney disease in Central America. Am J Kidney Dis 62:908–918. 10.1053/j.ajkd.2013.05.019 23850447 10.1053/j.ajkd.2013.05.019

[83] Sasai F, Rogers KL, Orlicky DJ, Stem A, Schaeffer J, Garcia G, Fox J, Ray MS, Butler-Dawson J, Gonzalez-Quiroz M, Leiva R, Taduri G, Anutrakululchai S, Venugopal V, Madero M, Glaser J, Wijkstrom J, Wernerson A, Brown JM, Johnson RJ, Roncal-Jimenez CA (2022) Inhaled silica nanoparticles cause chronic kidney disease in rats. Am J Physiol Renal Physiol 323:F48-f58. 10.1152/ajprenal.00021.2022 35635324 10.1152/ajprenal.00021.2022 PMC9236867

[84] Laws RL, Brooks DR, Amador JJ, Weiner DE, Kaufman JS, Ramírez-Rubio O, Riefkohl A, Scammell MK, López-Pilarte D, Sánchez JM, Parikh CR, McClean MD (2016) Biomarkers of kidney injury among Nicaraguan sugarcane workers. Am J Kidney Dis 67:209–217. 10.1053/j.ajkd.2015.08.022 26454687 10.1053/j.ajkd.2015.08.022 PMC4801230

[85] Butler-Dawson J, Dally M, Johnson RJ, Johnson EC, Krisher L, Sánchez-Lozada LG, Griffin BR, Brindley S, Newman LS (2020) Association of copeptin, a surrogate marker of arginine vasopressin, with decreased kidney function in sugarcane workers in Guatemala. Ann Nutr Metab 76:30–36. 10.1159/000506619 32172243 10.1159/000506619 PMC7212520

[86] Anastasio P, Cirillo M, Spitali L, Frangiosa A, Pollastro RM, De Santo NG (2001) Level of hydration and renal function in healthy humans. Kidney Int 60:748–756. 10.1046/j.1523-1755.2001.060002748.x 11473658 10.1046/j.1523-1755.2001.060002748.x

[87] El Boustany R, Tasevska I, Meijer E, Kieneker LM, Enhörning S, Lefèvre G, Mohammedi K, Marre M, Fumeron F, Balkau B, Bouby N, Bankir L, Bakker SJ, Roussel R, Melander O, Gansevoort RT, Velho G (2018) Plasma copeptin and chronic kidney disease risk in 3 European cohorts from the general population. JCI Insight 3:e121479. 10.1172/jci.insight.121479 29997293 10.1172/jci.insight.121479 PMC6124520

[88] Enhörning S, Bankir L, Bouby N, Struck J, Hedblad B, Persson M, Morgenthaler NG, Nilsson PM, Melander O (2013) Copeptin, a marker of vasopressin, in abdominal obesity, diabetes and microalbuminuria: the prospective Malmö Diet and Cancer Study cardiovascular cohort. Int J Obes (Lond) 37:598–603. 10.1038/ijo.2012.88 22614056 10.1038/ijo.2012.88

[89] Amaerjiang N, Li M, Xiao H, Zunong J, Li Z, Huang D, Vermund SH, Pérez-Escamilla R, Jiang X, Hu Y (2022) Dehydration status aggravates early renal impairment in children: a longitudinal study. Nutrients 14:335. 10.3390/nu14020335 35057516 10.3390/nu14020335 PMC8778530

[90] Zittema D, Casteleijn NF, Bakker SJ, Boesten LS, Duit AA, Franssen CF, Gaillard CA, Gansevoort RT (2017) Urine concentrating capacity, vasopressin and copeptin in ADPKD and IgA nephropathy patients with renal impairment. PLoS One 12:e0169263. 10.1371/journal.pone.0169263 28081165 10.1371/journal.pone.0169263 PMC5231267

[91] Kitiwan BK, Vasunilashorn SM, Baer HJ, Mukamal K, Juraschek SP (2021) The association of urine osmolality with decreased kidney function and/or albuminuria in the United States. BMC Nephrol 22:306. 10.1186/s12882-021-02478-9 34507548 10.1186/s12882-021-02478-9 PMC8434733

[92] Gansevoort RT, van Gastel MDA, Chapman AB, Blais JD, Czerwiec FS, Higashihara E, Lee J, Ouyang J, Perrone RD, Stade K, Torres VE, Devuyst O (2019) Plasma copeptin levels predict disease progression and tolvaptan efficacy in autosomal dominant polycystic kidney disease. Kidney Int 96:159–169. 10.1016/j.kint.2018.11.044 30898339 10.1016/j.kint.2018.11.044 PMC6640141

[93] Hebert LA, Greene T, Levey A, Falkenhain ME, Klahr S (2003) High urine volume and low urine osmolality are risk factors for faster progression of renal disease. Am J Kidney Dis 41:962–971. 10.1016/s0272-6386(03)00193-8 12722030 10.1016/s0272-6386(03)00193-8

[94] Torres VE, Grantham JJ, Chapman AB, Mrug M, Bae KT, King BF Jr, Wetzel LH, Martin D, Lockhart ME, Bennett WM, Moxey-Mims M, Abebe KZ, Lin Y, Bost JE (2011) Potentially modifiable factors affecting the progression of autosomal dominant polycystic kidney disease. Clin J Am Soc Nephrol 6:640–647. 10.2215/cjn.03250410 21088290 10.2215/CJN.03250410 PMC3082424

[95] Plischke M, Kohl M, Bankir L, Shayganfar S, Handisurya A, Heinze G, Haas M (2014) Urine osmolarity and risk of dialysis initiation in a chronic kidney disease cohort—a possible titration target? PLoS One 9:e93226. 10.1371/journal.pone.0093226 24675963 10.1371/journal.pone.0093226 PMC3968127

[96] Lee MJ, Chang TI, Lee J, Kim YH, Oh KH, Lee SW, Kim SW, Park JT, Yoo TH, Kang SW, Choi KH, Ahn C, Han SH (2019) Urine osmolality and renal outcome in patients with chronic kidney disease: results from the KNOW-CKD. Kidney Blood Press Res 44:1089–1100. 10.1159/000502291 31505490 10.1159/000502291

[97] Velho G, El Boustany R, Lefèvre G, Mohammedi K, Fumeron F, Potier L, Bankir L, Bouby N, Hadjadj S, Marre M, Roussel R (2016) Plasma copeptin, kidney outcomes, ischemic heart disease, and all-cause mortality in people with long-standing type 1 diabetes. Diabetes Care 39:2288–2295. 10.2337/dc16-1003 27729425 10.2337/dc16-1003

[98] Velho G, Ragot S, El Boustany R, Saulnier PJ, Fraty M, Mohammedi K, Fumeron F, Potier L, Marre M, Hadjadj S, Roussel R (2018) Plasma copeptin, kidney disease, and risk for cardiovascular morbidity and mortality in two cohorts of type 2 diabetes. Cardiovasc Diabetol 17:110. 10.1186/s12933-018-0753-5 30071874 10.1186/s12933-018-0753-5 PMC6071392

[99] Clark WF, Sontrop JM, Huang SH, Gallo K, Moist L, House AA, Cuerden MS, Weir MA, Bagga A, Brimble S, Burke A, Muirhead N, Pandeya S, Garg AX (2018) Effect of coaching to increase water intake on kidney function decline in adults with chronic kidney disease: the CKD WIT randomized clinical trial. JAMA 319:1870–1879. 10.1001/jama.2018.4930 29801012 10.1001/jama.2018.4930 PMC6583759

[100] Flahault A, Bollée G, El-Jalbout R, Cloutier A, Santos RAS, Lapeyraque AL, Luu TM, Nuyt AM (2022) Plasma copeptin is increased and associated with smaller kidney volume in young adults born very preterm. Clin Kidney J 15:709–717. 10.1093/ckj/sfab226 35371457 10.1093/ckj/sfab226 PMC8967663

[101] Vandergheynst F, Decaux G (2008) Lack of elevation of urinary albumin excretion among patients with chronic syndromes of inappropriate antidiuresis. Nephrol Dial Transplant 23:2399–2401. 10.1093/ndt/gfn124 18400823 10.1093/ndt/gfn124

[102] Watso JC, Farquhar WB (2019) Hydration status and cardiovascular function. Nutrients 11:1866. 10.3390/nu11081866 31405195 10.3390/nu11081866 PMC6723555

[103] Dmitrieva NI, Burg MB (2015) Elevated sodium and dehydration stimulate inflammatory signaling in endothelial cells and promote atherosclerosis. PLoS One 10:e0128870. 10.1371/journal.pone.0128870 26042828 10.1371/journal.pone.0128870 PMC4456159

[104] Arnaoutis G, Kavouras SA, Stratakis N, Likka M, Mitrakou A, Papamichael C, Sidossis LS, Stamatelopoulos K (2017) The effect of hypohydration on endothelial function in young healthy adults. Eur J Nutr 56:1211–1217. 10.1007/s00394-016-1170-8 26864199 10.1007/s00394-016-1170-8

[105] Pross N (2017) Effects of dehydration on brain functioning: a life-span perspective. Ann Nutr Metab 70(Suppl 1):30–36. 10.1159/000463060 28614811 10.1159/000463060

[106] Drozdowska A, Falkenstein M, Jendrusch G, Platen P, Luecke T, Kersting M, Jansen K (2020) Water consumption during a school day and children’s short-term cognitive performance: the CogniDROP randomized intervention trial. Nutrients 12:1297. 10.3390/nu12051297 32370147 10.3390/nu12051297 PMC7282257

[107] EFSA Panel on Dietetic Products, Nutrition, and Allergies (NDA) (2010) Scientific opinion on dietary reference values for water. EFSA J. 10.2903/j.efsa.2010.14592010

[108] Yamada Y, Zhang X, Henderson MET, Sagayama H, Pontzer H, Watanabe D, Yoshida T, Kimura M, Ainslie PN, Andersen LF, Anderson LJ, Arab L, Baddou I, Bedu-Addo K, Blaak EE, Blanc S, Bonomi AG, Bouten CVC, Bovet P, Buchowski MS, Butte NF, Camps SG, Close GL, Cooper JA, Cooper R, Das SK, Dugas LR, Eaton S, Ekelund U, Entringer S, Forrester T, Fudge BW, Goris AH, Gurven M, Halsey LG, Hambly C, El Hamdouchi A, Hoos MB, Hu S, Joonas N, Joosen AM, Katzmarzyk P, Kempen KP, Kraus WE, Kriengsinyos W, Kushner RF, Lambert EV, Leonard WR, Lessan N, Martin CK, Medin AC, Meijer EP, Morehen JC, Morton JP, Neuhouser ML, Nicklas TA, Ojiambo RM, Pietiläinen KH, Pitsiladis YP, Plange-Rhule J, Plasqui G, Prentice RL, Rabinovich RA, Racette SB, Raichlen DA, Ravussin E, Redman LM, Reilly JJ, Reynolds RM, Roberts SB, Schuit AJ, Sardinha LB, Silva AM, Sjödin AM, Stice E, Urlacher SS, Valenti G, Van Etten LM, Van Mil EA, Wells JCK, Wilson G, Wood BM, Yanovski JA, Murphy-Alford AJ, Loechl CU, Luke AH, Rood J, Westerterp KR, Wong WW, Miyachi M, Schoeller DA, Speakman JR (2022) Variation in human water turnover associated with environmental and lifestyle factors. Science 378:909–915. 10.1126/science.abm8668 36423296 10.1126/science.abm8668 PMC9764345

