# Supplementary material for: Is mild dehydration a risk for progression of childhood chronic kidney disease?
Source: Pediatr Nephrol. 2024 Apr 18;39(11):3177–91. doi: 10.1007/s00467-024-06332-6 (PMC11413076; doi:10.1007/s00467-024-06332-6)
Supplement: Supplementary file 1 — Graphical abstract (PPTX 136 KB) [file 467_2024_6332_MOESM1_ESM.pptx]

## Slide 1
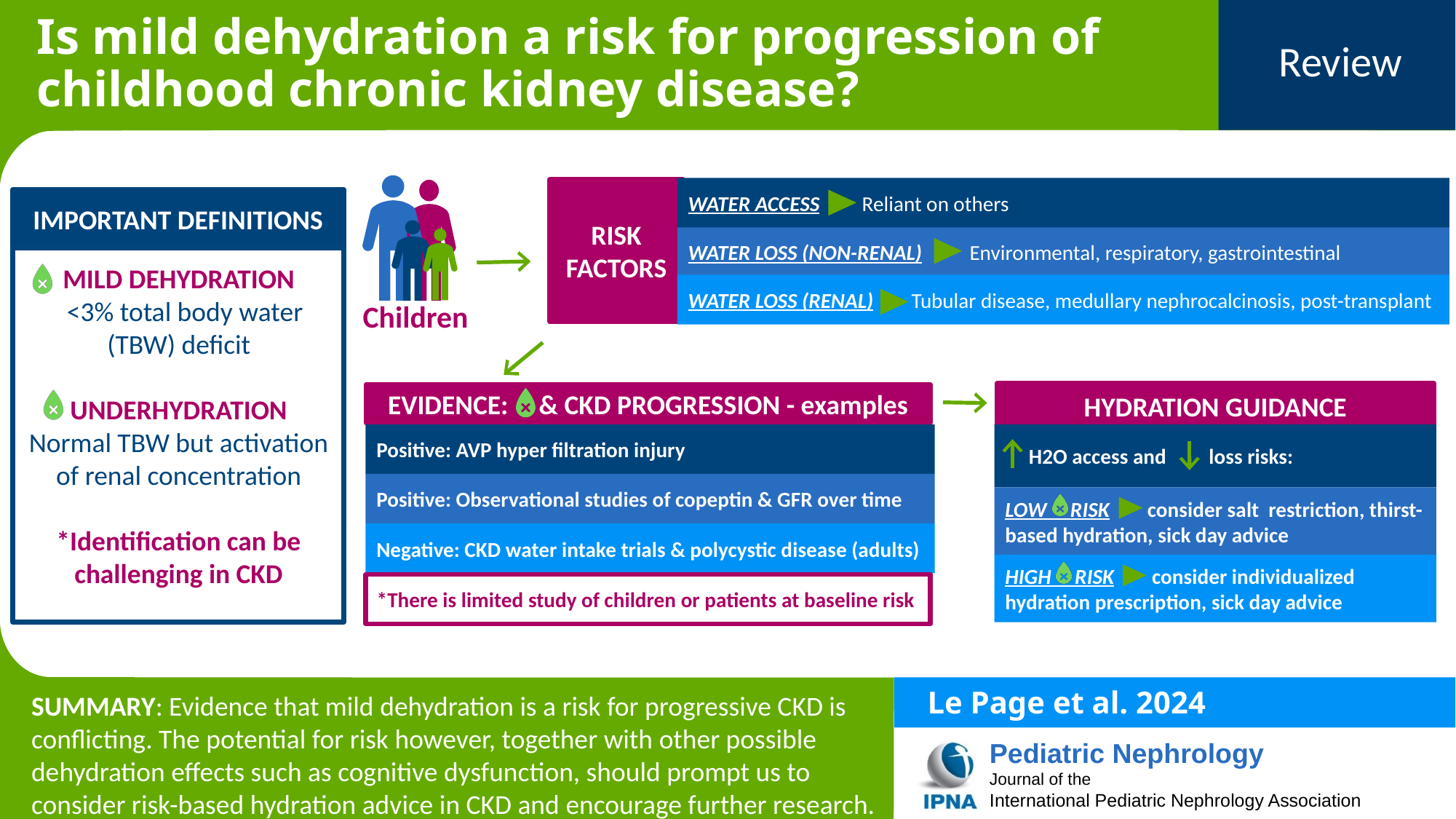

Is mild dehydration a risk for progression of childhood chronic kidney disease?
WATER ACCESS Reliant on others
RISK FACTORS
IMPORTANT DEFINITIONS
WATER LOSS (NON-RENAL) Environmental, respiratory, gastrointestinal
MILD DEHYDRATION
 <3% total body water (TBW) deficit
UNDERHYDRATION
Normal TBW but activation of renal concentration
*Identification can be challenging in CKD
WATER LOSS (RENAL) Tubular disease, medullary nephrocalcinosis, post-transplant
Children
HYDRATION GUIDANCE
EVIDENCE: & CKD PROGRESSION - examples
Positive: AVP hyper filtration injury
 H2O access and loss risks:
Positive: Observational studies of copeptin & GFR over time
LOW RISK consider salt restriction, thirst-based hydration, sick day advice
Negative: CKD water intake trials & polycystic disease (adults)
HIGH RISK consider individualized hydration prescription, sick day advice
*There is limited study of children or patients at baseline risk
Le Page et al. 2024
SUMMARY: Evidence that mild dehydration is a risk for progressive CKD is conflicting. The potential for risk however, together with other possible dehydration effects such as cognitive dysfunction, should prompt us to consider risk-based hydration advice in CKD and encourage further research.
